# Activation of Mammary Epithelial and Stromal Fibroblasts upon Exposure to *Escherichia coli* Metabolites

**DOI:** 10.3390/cells13201723

**Published:** 2024-10-17

**Authors:** Jamilah H. Alshehri, Huda K. Al-Nasrallah, Mysoon M. Al-Ansari, Abdelilah Aboussekhra

**Affiliations:** 1 Department of Molecular Oncology, King Faisal Specialist Hospital and Research Center, Riyadh 11211, Saudi Arabia; 2 Department of Botany and Microbiology, College of Science, King Saud University, Riyadh 11451, Saudi Arabia

**Keywords:** breast cancer, cancer-associated fibroblasts, *E. coli*, microbiota, EMT

## Abstract

Breast cancer is the leading cause of cancer death among women worldwide. The mammary gland is composed of various types of cells including luminal cells, fibroblasts, immune cells, adipocytes, and specific microbiota. The reciprocal interaction between these multiple types of cells can dictate the initiation and progression of cancer, as well as metastasis and response to therapy. In the present report, we have shown that *Escherichia coli*-conditioned media (E-CM) can directly activate human mammary luminal epithelial cells (HMLEs), by inducing epithelial-to-mesenchymal transition (EMT), a process associated with increased proliferation and invasion capacities, as well as stemness features. Additionally, it has been shown that E-CM has an indirect pro-carcinogenic effect, mediated by the activation of normal breast fibroblasts (NBFs). Indeed, E-CM upregulated various markers of active fibroblasts (FAP-α, GPR77, and CD10), and enhanced the proliferation, migration, and invasion capacities of NBFs. Furthermore, E-CM induced an inflammatory response in NBFs by activating the pro-inflammatory NF-kB transcription factor and several of its downstream target cytokines including IL-1β, IL-6, and IL-8. This E-CM-dependent activation of NBFs was confirmed by showing their paracrine pro-carcinogenic effects through inducing EMT and stemness features in normal breast epithelial cells. Interestingly, similar effects were obtained by recombinant human IL-1β. These results provide the first indication that *E. coli* can initiate breast carcinogenesis through the activation of breast stromal fibroblasts and their paracrine pro-carcinogenic effects.

## 1. Introduction

Breast cancer is the most prevalent and leading cause of cancer death among women worldwide [1]. When invasive, breast carcinomas are composed of cancer cells surrounded by several other types of cells, which, along with noncellular components, constitute the tumor microenvironment (TME) [2,3]. Cancer-associated fibroblasts (CAFs), the most active type of cells in breast carcinomas, promote carcinogenesis through the secretion of high levels of several pro-carcinogenic cytokines such as SDF-1, TGF-β1, and IL-6 [4,5]. These cancer-promoting factors are under the control of two important transcription regulatory pathways, NF-kB and STAT3. The transactivation of breast stromal fibroblasts could result from crosstalk with cancer cells, or other types of cells present in the TME, such as bacteria. Indeed, it has become clear that the mammary gland, which has traditionally been considered sterile, has its own specific microbiota, distinct from that found at other body sites [6,7]. Urbaniak et al. identified eight species and seven bacterial phyla in breast tissue samples. *Proteobacteria* and *Firmicutes* (specifically the class *Bacilli*) are the most abundant phyla in breast tissue [7]. Recently, intracellular microorganisms have been identified in breast tissue [8,9]. The imbalance of the microbiome, in which harmful bacteria outcompete the normal bacteria (dysbiosis), may contribute to the formation of carcinomas [10,11]. Although it has not yet been proven that dysbiosis causes breast cancer, breast tissue samples show differences in the composition and abundance of some specific bacterial taxa between breast cancer patients and healthy controls. In general, women with breast cancer have a higher relative abundance of *Escherichia coli* (*E. coli*) than healthy controls [7,12]. Additionally, probes detecting 16S rRNA confirmed the presence of *E. coli* in triple-negative breast cancer tissues, and *E. coli* isolated from the normal adjacent tissue of breast cancer patients was able to induce double-strand DNA breaks in HeLa cells [7,13].

Therefore, in the present study, we sought to investigate the potential role of *E. coli* (a component of the breast microenvironment) in promoting breast carcinogenesis by affecting both epithelial cells and their stromal fibroblasts. We found that *E. coli* cells have the capacity to transactivate breast epithelial cells and their stromal fibroblasts in a paracrine manner.

## 2. Materials and Methods

### 2.1. Cells and Cell Culture

Normal breast fibroblasts were isolated from healthy women who had undergone mammoplasty at KFSH&RC as previously described [14]. All patients provided consent under an Research Advisory Council (RAC) approved proposal #2,031,091 by the institutional ethics committee. Fibroblasts were cultured using fibroblast growth medium 199, Ham’s F12, supplemented with 20% fetal bovine serum (FBS) and 1% antibiotic antimycotic (ABM) (Gibco, Grand Island, NY, USA). Human mammary luminal epithelial cells (HMLEs) were cultured in a DMEM/F-12 medium, with Bovine Pituitary Extract (BPE), HuMEC, 1% ABM, and 2% FBS (Gibco, Grand Island, NY, USA). MCF-7 cells were cultured in a RPMI medium supplemented with 10% FBS and 1% AB. All cells were cultured at 37 °C and 5% CO_2_ in a humidified incubator. Pure recombinant human IL-1β (rhIL-1β) was purchased from Abcam (Cambridge, UK).

### 2.2. Fibroblast-Conditioned Medium

Treated fibroblast cells were re-cultured in a serum-free medium (0.5% FBS) for 24 h, and then the resulting conditioned medium (SFCM) was collected and centrifuged at 1500 rpm for 5 min at 4 °C. The supernatants were either used immediately or stored for further use at −80 °C.

### 2.3. Bacteria-Conditioned Medium

*E. coli* (DH10B) was cultured in Luria–Bertani broth (LB) at 37 °C on an orbital shaker at 200 rpm for 24 h. The cultured medium was collected and centrifuged at 4000 rpm for 10 min at 4 °C. The obtained bacterial culture supernatant (hereafter referred to as “conditioned medium”; CM) was filtered using a 0.10 µm Stericup vacuum filter (Merck, Darmstadt, Germany) and stored at −80 °C.

### 2.4. Purification of RNA and qRT-PCR

Total RNA was purified using the RNeasy mini kit (QIAGEN, Manchester, UK, 74104), following the manufacturer’s guidelines. RNA concentration was measured using the Nanodrop-1000 Spectrophotometer (Thermo Fisher, Waltham, MA, USA). Complementary DNA (cDNA) was obtained from reverse transcription of 1 µg of RNA using the Advantage RT-PCR Kit (Catalog number: 639505; Clontech Laboratories, Mountain View, CA, USA) following the guidelines of the manufacturer. For quantitative RT-PCR (qRT-PCR), FastStart Essential DNA Green Master 2X (Roche, New York, NY, USA, 6402712001) and primers at 1.66 μM final concentration were used. GAPDH was utilized as the endogenous control gene. All reactions were performed in triplicate, and the obtained data were analyzed using the delta CT method. The sequences of the utilized primers are shown in Table 1:

### 2.5. NF-kB RT-PCR Array

The RT^2^ Profiler PCR Array Format F (Human NF-kB Signaling Targets) (QIAGEN, PAHS-225ZA) was used according to the manufacturer’s instructions. As recommended, 500 ng of pure RNA was used to make cDNA. 100 μL of the cDNA synthesis reaction was diluted in RNase-free water and then mixed with FastStart Essential DNA Green Master 2X. A total of 25 μL of master mix was added to each well of the RT^2^ Profiler PCR Array.

### 2.6. Preparation of Cellular Lysates and Immunoblotting

The preparation of cellular lysates and immunoblotting was performed as previously described [15]. Antibodies against α-SMA (Ab5694), FAP-α (Ab53066), IL-1β (Ab2105), IL-6 (Ab6672), IL-8 (Ab106350), Twist-1 (Ab175430), and Vimentin (Ab8978) were purchased from Abcam (Cambridge, UK). ALDH-1 (611195) was purchased from BD Biosciences (USA). BMI-1 (6964), E-cadherin (14472), EpCAM (2929), MMP-2 (4022), N-cadherin (14215), p-NF-kB p65 (3031), p-STAT3 (9145), Snail (3879), and STAT-3 (9139) were purchased from Cell Signaling Technology (Danvers, MA, USA). CD10 (Sc-46656), GAPDH (Sc-25778), GPR77 (Sc-515734), and NF-kB p65 (Sc-8008) were purchased from Santa Cruz (Santa Cruz, CA, USA). CD44 was purchased from Sigma-Aldrich (St. Louis, MI, USA).

### 2.7. Quantification of Protein Levels 

Protein levels were determined using the software ImageJ (v 1.53) (NIH). Protein levels were normalized to the internal control GAPDH, and fold change was determined after comparison with the control sample.

### 2.8. Proliferation, Migration, and Invasion Assays

The Real-Time Cell Analyzer–Dual Plat (RTCA-DP) xCELLigence System (ACEA Biosciences, Santa Clara, CA, USA) was used to determine the proliferation, migration, and invasion capacities of the cells. In the upper chamber of the CIM-plate, cells (1 × 10^4^) were cultured in a serum-free medium (SFM) and coated in Matrigel (BD Biosciences) diluted in SFM (1:30 for invasion) or non-coated (for migration) to gravitate toward the chemoattractant located in the lower chamber. For the proliferation assay, cells (1 × 10^4^) were seeded in the E-plate with a complete medium following the manufacturer’s guidelines. All the experiments were performed in triplicates.

### 2.9. Enzyme-Linked Immunosorbent Assay (ELISA)

The conditioned media of the treated breast fibroblasts were collected after 24 h of treatment and briefly centrifuged to assess the levels of secreted IL-1β (ab46052; Abcam), IL-6 (D6050; R&D Systems, Minneapolis, MN, USA), and IL-8 (D8000C; R&D Systems) following the guidelines of the manufacturers. The optical density was measured at 570 nm, and the secreted protein concentrations were analyzed. These experiments were repeated at least 3 times.

### 2.10. Human Cytokine Antibody Array

The conditioned media of the breast fibroblasts were applied to the Human Cytokine Array C5 (AAH-CYT-5-8; RayBiotech, Georgia, GA, USA) according to the manufacturer’s instructions. Quantitative array analysis was performed using ImageJ and presented as a heatmap using the GraphPad Prism software (version 9).

### 2.11. Spheroid Formation Assay

HMLE cells (2000 per well) were seeded in an ultra-low attachment 96-well plate and cultured in a stem cell-specific medium composed of DMEM/F12, 4% FBS, 1% ABM, 1% GlutaMax, 2% B-27 (Gibco), hEGF, hbFGF, hydrocortisone, insulin, and heparin sodium salt (Sigma-Aldrich). Cells were incubated for 7 days at 37 °C and 5% CO_2_. The number of formed mammospheres upon mechanical dissociation with a 100 µm diameter was counted and imaged using a fluorescence microscope (FLoid Cell Imaging Station, Washington, DC, USA).

### 2.12. Statistical Analysis

Statistical analysis of the data was achieved using the software GraphPad Prism. Two-tailed unpaired Student’s *t*-test was utilized for continuous numerical analysis, and *p*-values of 0.05 or less were considered statistically significant.

## 3. Results

### 3.1. E. coli Metabolites Activate Breast Stromal Fibroblasts

**Figure 1:**
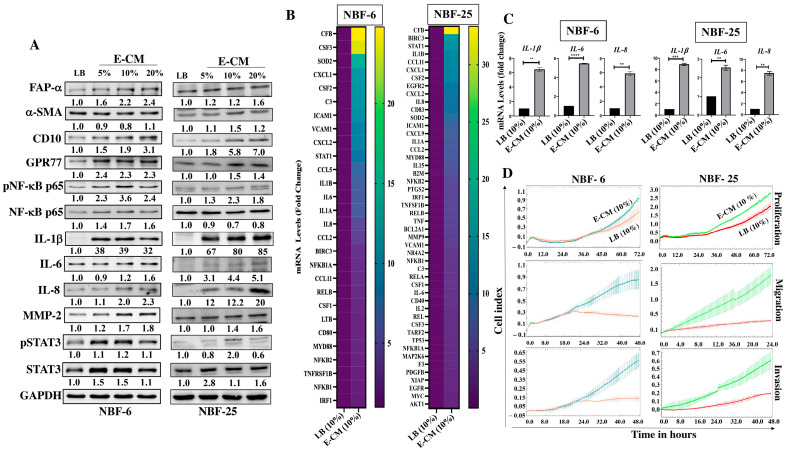
*E. coli*-conditioned medium activates breast stromal fibroblasts. Normal breast fibroblasts (NBF-6 and NBF-25) were treated with LB (10%) as a control or E-CM (5%, 10%, and 20%) for 24 h. (**A**) Cellular lysates were prepared and used for immunoblotting analysis using specific antibodies against the indicated proteins. The values beneath the bands represent the fold change in the intensity of bands compared with the control sample after correction against the internal control GAPDH. Phospho-proteins were further normalized to their corresponding non-phosphorylated proteins. (**B**) Total RNA was extracted from the indicated cells treated with LB (10%) or E-CM (10%) for 24 h, and 0.5 μg of total RNA was used for reverse transcription. PCR component mixture (25 μL) was added to each well of the RT^2^ Profiler PCR Array specific for human NF-kB signaling targets (PAHS-225ZA). Gene expression levels were normalized to the internal control GAPDH and presented in the viridis heatmap. Columns represent samples, and rows represent each gene. Each cell in the heatmap represents the expression level of each gene by fold change. (**C**) NBF-6 and NBF-25 cells were treated with LB (10%) or E-CM (10%) for 24 h. Total RNA was isolated, and the mRNA expression levels of the indicated genes were assessed by qRT-PCR using specific primers and then normalized against the internal control GAPDH. Error bars represent the mean ± SEM, (n = 3). ** *p* ≤ 0.01, *** *p* ≤ 0.001, **** *p* ≤ 0.0001. (**D**) The proliferation, migration, and invasion capacities of NBF-6 and NBF-25 cells treated with LB (10%) or E-CM (10%) were evaluated using the RTCA-DP xCELLigence System for the indicated periods of time. Data are representative of different experiments performed in triplicate.

To assess the effect of the *E. coli* secretome on normal breast fibroblasts, NBF-6 and NBF-25 cells were treated either with LB (10%) and used as a control or with a conditioned medium (E-CM) (5%, 10%, and 20%) for 24 h. Whole cell lysates were prepared, and specific antibodies were used for immunoblotting analysis. Figure 1A shows the E-CM-related increase in the level of the fibroblast activation marker FAP-a in a dose-dependent manner in both cell cultures (NBF-6 and NBF-25). The other important CAF biomarker α-SMA was also upregulated (1.5-fold in response to 10% E-CM in NBF-25) but not in NBF-6 cells. Additionally, for both NBF-6 and NBF-25 cells, E-CM upregulated CD10 (in a concentration-dependent manner) and GPR77 (2.4-fold in response to 5% E-CM and 1.5-fold in response to 10% E-CM, respectively) (Figure 1A). CD10 and GPR77 are two other important markers of active fibroblasts [16]. Interestingly, the transcription factor NF-kB was activated in both NBF-6 and NBF-25 cells in response to E-CM (10%, which was chosen as the optimal concentration for further analysis) (Figure 1A). For both NBF-6 and NBF-25, the activation of NF-kB was confirmed by showing a strong upregulation of its downstream target IL-1b (39-fold and 80-fold in response to 10% E-CM, respectively), as well as IL-6 and IL-8 (Figure 1A). To confirm E-CM-dependent activation of the NF-kB pathway, NBF-6 and NBF-25 cells were treated with LB (10%) or E-CM (10%) for 24 h, and the RT2 Profiler PCR Array specific for human NF-kB signaling targets was performed to test the effect of E-CM (10%) on NF-kB target genes. Relative to controls, treatment with E-CM affected the expression (more than 1.5-fold) of 28 genes in NBF-6 cells and 48 genes in NBF-25 cells (Figure 1B). The commonly differentially expressed genes (24) are depicted in Table 1. These genes include transcription factors such as NF-kB and *STAT1* as well as many pro-inflammatory factors such as *IL-6*, *IL-8*, *IL-1β*, *CXCL2*, and *CCL-2* (Table 2). The CFB gene showed the strongest differential expression in both NBF-6 and NBF-25 (24.8 and 33.4, respectively) followed by *CXCL1*, *CSF2*, and *CXCL2*, which were upregulated more than 10-fold in both cell cultures upon treatment with E-CM (Table 1). These findings confirm that *E. coli*-secreted metabolites activate the NF-kB inflammatory pathway in NBFs. To confirm these findings, we tested by qRT-PCR the effect of E-CM on the mRNA level of the pro-inflammatory cytokines *IL-1β*, *IL-6*, and *IL-8* in NBF-6 and NBF-25 cells treated as detailed above. Figure 1C shows that for both NBF-6 and NBF-25 cells, *IL-1β* expression increased 6- and 9-folds, respectively; *IL-6* expression increased 5- and 2.5-folds, respectively; and *IL-8* expression increased 6- and 7.5-fold, respectively, in response to E-CM (10%) compared with LB (10%). These results confirm the PCR array findings and show that E-CM affects the expression of several cytokines at the mRNA level.

In order to show E-CM-dependent activation of breast stromal fibroblasts, the cellular proliferation, migration, and invasion capacities of NBF-6 and NBF-25 cells treated with E-CM (10%) or LB (10%) were assessed using the RTCA-DP xCELLigence system. Figure 1D shows an increase in the proliferation rate of both cell cultures treated with E-CM (10%) compared with their respective controls. This increased proliferation rate was confirmed at the protein level by showing activation of the transcription factor STAT3 (Figure 1A). Additionally, Figure 1D shows an increase in the migratory and the invasive capacities of NBF-6 and NBF-25 cells treated with E-CM (10%) compared with controls. This increase in the invasive abilities was confirmed at the protein level by showing a dose-dependent increase in the level of MMP-2 (Figure 1A), a well-known pro-invasive protein [17].

### 3.2. E. coli-Metabolite-Activated Normal Breast Fibroblasts Secrete High Levels of Several Pro-Carcinogenic Cytokines

**Figure 2:**
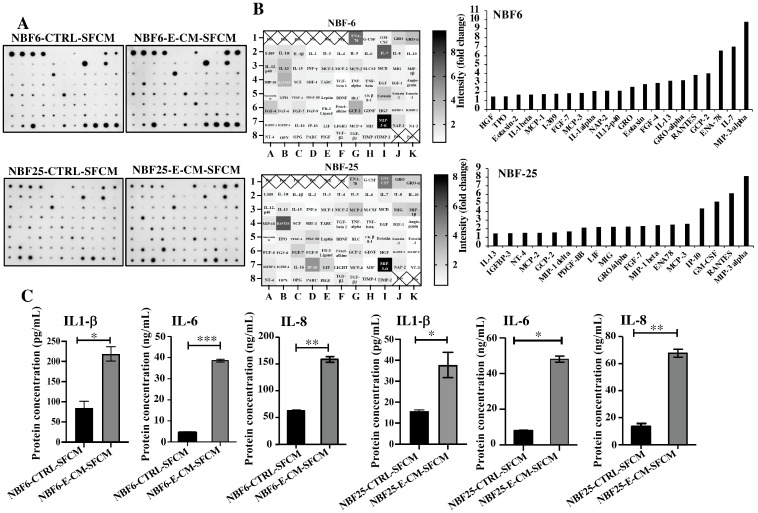
*E. coli*-conditioned medium-activated normal breast fibroblasts secrete high levels of pro-carcinogenic cytokines. NBF-6 and NBF-25 cells were treated with LB (10%) as a control or E-CM (10%) for 24 h, and then were re-incubated in the presence of SFM for another 24 h. The resulting SFCM (NBF6-CTRL-SFCM and NBF6-E-CM-SFCM, respectively) were collected. (**A**) SFCM were applied to the RayBiotech Human Cytokine Array C5. The spots correspond to different cytokines. (**B**) The intensities of the spots in the cytokine array were quantified by densitometric analysis, normalized to the density of the positive controls of each membrane (POS), and presented as fold changes. Left panels: single gradient heatmaps; right panels: histograms showing the intensities of the differentially secreted cytokines (>1.5-fold). (**C**) The levels of the secreted proteins were assessed by ELISA in the indicated SFCM and presented in the respective histograms. Error bars represent the mean ± SEM (n = 3). * *p* ≤ 0.05, ** *p* ≤ 0.01, *** *p* ≤ 0.001.

In order to investigate the effect of E-CM on the secretion of cytokines, NBF-6 and NBF-25 cells were treated with E-CM (10%) or LB (10%) for 24 h, and then cells were reincubated in a serum-free medium for another 24 h, and then the resulting serum-free conditioned media (SFCM) were collected and applied to the Human Cytokine Antibody Array (Figure 2A). The intensities of the spots were quantified by densitometry, and the obtained results are presented in the heatmap as fold changes relative to LB-treated cells, while the genes with a differential expression equal/above 1.5-fold were depicted in a histogram (Figure 2B). The cytokine array analysis showed an increase in the pro-carcinogenic and -inflammatory cytokines MIP-3-α, ENA-78, GCP-2, RANTES, IL-13, GRO-α, FGF-4, Eotaxin, GRO, NAP-2, IL-1α, MCP-3, FGF-7, I-309, MCP-1, IL-1β, Eotaxin-2, TPO, and HGF (Figure 2B). Interestingly, seven cytokines were differentially expressed in both NBF-6 and NBF-25: MIP-3α, RANTES, MCP-3, ENA78, GROα, GCP-2, and IL-13 (Figure 2B). MIP-3α showed the highest differential expression (about 10-fold) in both cell cultures (Figure 2B). These results were confirmed using ELISA for the cytokines IL-1β, IL-6, and IL-8. Indeed, treatment of NBF-6 and NBF-25 cells with E-CM (10%) significantly increased the secretion levels of these three cytokines compared with their levels secreted from LB-treated cells (Figure 2C). These results indicate *E. coli*-dependent activation of normal breast fibroblasts, in addition to a strong pro-inflammatory response through activation of the pro-inflammatory signaling pathways NF-kB and STAT3.

### 3.3. E. coli-Metabolites-Activated Normal Breast Fibroblasts Induce EMT and Stemness in Normal Breast Epithelial Cells in a Paracrine Manner

**Figure 3:**
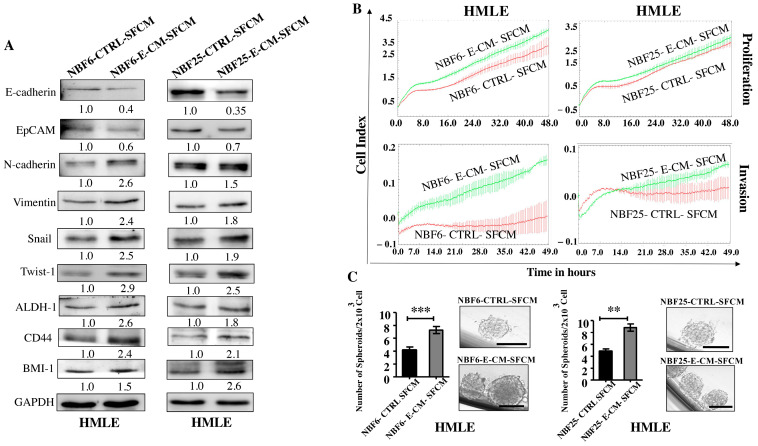
*E. coli*-conditioned medium-activated normal breast fibroblasts induce EMT and stemness in normal breast epithelial cells in a paracrine manner. NBF-6 and NBF-25 cells were treated with LB (10%) as a control or E-CM (10%) for 24 h, and then were re-incubated in the presence of SFM for another 24 h. SFCM (NBF6-CTLRL-SFCM and NBF6-E-CM-SFCM, respectively) were collected and used to treat HMLE cells for 24 h. (**A**) Cellular lysates were prepared and used for immunoblotting analysis using specific antibodies against the indicated proteins. The values beneath bands represent the fold change of the intensity of the bands compared with the control sample after correction against the internal control GAPDH. (**B**) The proliferation and invasion capacities of HMLE cells treated with the indicated SFCM were assessed using the RTCA-DP xCELLigence system for the indicated periods of time. The data are representative of different experiments performed in triplicate. (**C**) HMLE cells (2 × 10^3^) treated with the indicated SFCM were seeded in an ultra-low attachment 96-well plate containing a stem cell-specific medium. The formed mammospheres were photographed, scale bar = 100 µm. Histograms represent the number of formed mammospheres (diameter ≥ 100 µm). Error bars represent mean ± SEM (n = 3). ** *p* ≤ 0.01, *** *p* ≤ 0.001.

The obtained results suggested that E-CM-activated normal breast fibroblasts could induce breast carcinogenesis in a paracrine manner. Therefore, NBF-6 and NBF-25 cells were treated with either LB (10%) as a control or with E-CM (10%) for 24 h, and then cells were washed and re-incubated in the presence of SFM for another 24 h. The obtained E-CM-free serum-free conditioned media (SFCM) were collected and used to treat normal human mammary luminal epithelial cells (HMLEs) for 24 h. Whole cell lysates were prepared, and specific antibodies were used for immunoblotting analysis. Figure 3A shows that NBF6-E-CM-SFCM and NBF25-E-CM-SFCM decreased the epithelial cell markers E-cadherin and EpCAM and increased the levels of the mesenchymal cell markers N-cadherin, Vimentin, Snail, and Twist-1 compared with their respective levels in control cells. Furthermore, NBF6-E-CM-SFCM and NBF25-E-CM-SFCM enhanced the proliferative and invasive capacities of HMLE cells compared with their corresponding controls (Figure 3B). This shows that E-CM-treated fibroblasts can induce EMT in breast luminal cells in a paracrine manner.

Next, we investigated the effect of NBF6-E-CM-SFCM and NBF25-E-CM-SFCM on the stemness features of HMLE cells. To this end, whole cell lysates were prepared from HMLE-treated cells with NBF6-E-CM-SFCM and NBF25-E-CM-SFCM and their corresponding controls, and specific antibodies against the stemness markers ALDH-1, BMI-1, and CD44 were used for immunoblotting analysis. Figure 3A shows an increase in the levels of ALDH-1 (2.6- and 1.8-fold, respectively), CD44 (2.4- and 2.1-fold, respectively), and BMI-1 (1.5- and 2.6-fold, respectively) in response to NBF6-E-CM-SFCM and NBF25-E-CM-SFCM compared to their levels in control cells. These changes in the expression of stemness-related biomarkers prompted us to investigate the ability of NBF6-E-CM-SFCM and NBF25-E-CM-SFCM to promote the formation of mammospheres in HMLE cells. Thereby, a single-cell suspension of HMLE cells (2 × 10^3^) treated with NBF6-E-CM-SFCM and NBF25-E-CM-SFCM and their corresponding controls were seeded in an ultra-low attachment 96-well plate containing a stem cell-specific culture medium and cultured for 7 days. The number of formed mammospheres with a diameter of ≥100 µm was significantly higher in HMLE cells treated with NBF6-E-CM-SFCM and NBF25-E-CM-SFCM than in their respective controls (Figure 3C). These results indicate that *E. coli*-activated normal breast fibroblasts have the capacity to promote the pro-carcinogenic processes EMT and stemness in normal breast epithelial cells in a paracrine manner. This demonstrates that *E. coli* metabolites activate breast stromal fibroblasts.

### 3.4. E. coli-Secreted Metabolites Induce EMT and Stemness in Normal Breast Epithelial Cells

**Figure 4:**
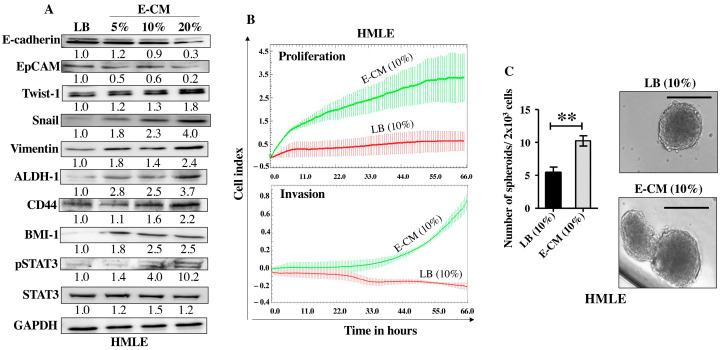
*E. coli*-conditioned medium induces EMT and stemness in normal breast epithelial cells. Normal breast epithelial cells (HMLEs) were treated with either LB (10%) as a control, or E-CM (5%, 10% and 20%) for 24 h. (**A**) Cellular lysates were prepared and used for immunoblotting analysis using specific antibodies against the indicated proteins. The values beneath the bands represent the fold change in the intensity of the bands compared with the control sample after correction against the internal control GAPDH. Phospho-proteins were further normalized to their corresponding non-phosphorylated proteins. (**B**) HMLE cells were treated with LB (10%) or E-CM (10%) and the proliferation and invasion rates were assessed using the RTCA-DP xCELLigence System. Data are representative of different experiments performed in triplicate. (**C**) HMLE cells (2 × 10^3^) treated with LB (10%), or E-CM (10%) were seeded in an ultra-low attachment 96-well plate containing a stem cell-specific medium. The formed mammospheres were photographed, scale bar = 100 µm. Histograms represent the number of formed mammospheres (diameter ≥ 100 µm). Error bars represent mean ± SEM (n = 3). ** *p* ≤ 0.01.

After showing the activation effects of *E. coli* on normal breast stromal fibroblasts, we decided to investigate the direct effect of *E. coli* on normal breast epithelial cells. To this end, HMLE cells were treated directly either with LB (10%) and used as a control or with E-CM (5%, 10% and 20%) for 24 h. Whole cell lysates were prepared and specific antibodies were used for immunoblotting analysis. While E-CM (20%) decreased the levels of the epithelial cell markers E-cadherin (3.3-fold) and EpCAM (5-folds), it increased the levels of the mesenchymal cell markers Twist-1 (1.8-fold), Snail (4-folds) and Vimentin (2.4-folds) relative to their levels in the control cells (Figure 4A). These changes in the expression of these genes suggested promotion of EMT in breast luminal cells. This was confirmed by showing strong increase in the invasive and proliferative capacities of HMLE cells treated with E-CM (10%) compared with the control (Figure 4B). This increased proliferation rate was confirmed at the protein level with concentration-dependent activation of the pro-carcinogenic transcription factor STAT3 (Figure 4A). The level of the active (phosphorylated) form of the protein was 4-folds and 10-folds higher in cells treated with E-CM relative to controls (Figure 4A). Since this signaling pathway plays a major role in stemness [18], we sought to assess the direct effect of E-CM on the stemness features of HMLE cells. Figure 4A shows an increase in the protein levels of the stemness markers ALDH-1 (3.7-fold in response to 20% E-CM), CD44 (in a concentration-dependent manner), and BMI-1 (2.5-fold in response to 10% and 20% E-CM) compared with the control (LB). This suggests E-CM-dependent promotion of stemness in HMLE cells. To confirm this, we decided to study the ability of E-CM to promote the formation of mammospheres in HMLE cells. Therefore, HMLE cells (2 × 10^3^) treated with LB (10%) or E-CM (10%) were seeded in an ultra-low attachment 96-well plate containing a stem cell-specific culture medium and cultured for 7 days. The number of formed mammospheres with a diameter ≥ 100 µm was significantly higher in HMLE cells treated with E-CM (10%) than in those treated with LB (Figure 4C). These findings indicate that *E. coli* metabolites can promote carcinogenesis through induction of the pro-carcinogenic processes EMT and stemness through activation of the STAT3 pathway in normal breast luminal cells.

### 3.5. Recombinant Human IL-1β Induces EMT and Stemness in Normal and Neoplastic Breast Epithelial Cells

**Figure 5:**
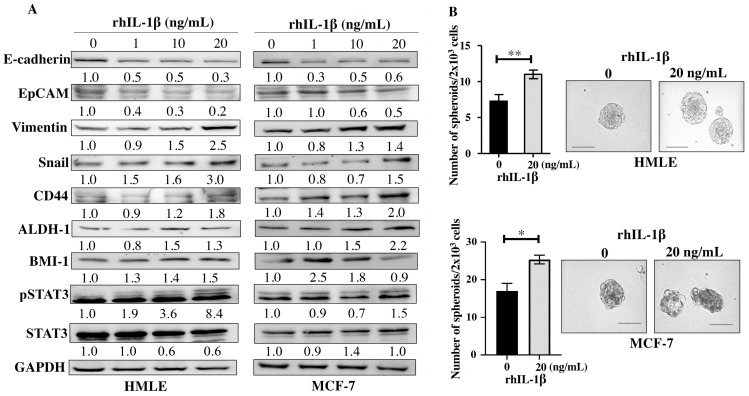
Recombinant human IL-1β induces EMT and stemness in normal breast epithelial cells and breast cancer cells. Exponential growing HMLE and MCF-7 cells were treated with increasing doses of rhIL-1β (0, 1, 10, and 20 ng/mL) for 24 h. (**A**) Western blot images. The values beneath the bands represent the fold change in the intensity of the bands compared with the control sample after correction against the internal control GAPDH. (**B**) HMLE cells (2 × 10^3^) either sham-treated (0) or challenged with rhIL-1β (20 ng/mL) were seeded in an ultra-low attachment 96-well plate containing a stem cell-specific medium. The formed spheres were photographed, scale bar = 100 µm. Histograms represent the number of formed mammospheres (diameter ≥ 100 µm). Error bars represent mean ± SEM (n = 3). * *p* ≤ 0.05, ** *p* ≤ 0.01.

Since the E-CM-activated normal breast fibroblasts secreted high levels of the pro-carcinogenic IL-1β, we sought to investigate the role of this cytokine in the induction and promotion of breast carcinogenesis. To achieve this, breast epithelial cells either normal (HMLE) or neoplastic (MCF-7) were treated with pure recombinant human IL-1β (rhIL-1β) (0, 1, 10 and 20 ng/mL) for 24 h. Whole cell lysates were prepared, and specific antibodies were used for immunoblotting analysis. For both HMLE and MCF-7 cells, rhIL-1β decreased the levels of the epithelial cell markers E-cadherin and EpCAM and increased the levels of the mesenchymal cell markers Vimentin (2.5- and 1.4-fold in response to 20 ng/mL, respectively) and Snail (3.0- and 1.5-fold in response to 20 ng/mL, respectively) (Figure 5A). This indicates that rhIL-1β promoted EMT in both normal (HMLE) and cancer (MCF-7) cells. Thereby, we decided to investigate the effect of rhIL-1β on the stemness features of breast epithelial cells. Figure 5A shows that rhIL-1β upregulated several breast cancer stem cell markers, namely CD44, ALDH-1, and BMI-1 in both HMLE and MCF-7 cells. Furthermore, rhIL-1β activated the stemness-related pathway STAT3 in both HMLE and MCF-7 cells (Figure 5A). This suggests rhIL-1β-dependent induction of stemness. To confirm this, we tested the ability of rhIL-1β to promote the formation of mammospheres. To this end, HMLE and MCF-7 cells (2 × 10^3^) were either sham-treated and used as a control or challenged with rhIL-1β (20 ng/mL), and then plated in a 96-well ultra-low attachment plate containing a stem cell-specific culture medium and cultured for 7 days. The presence of rhIL-1β significantly increased the number of formed mammospheres with a diameter ≥ 100 µm compared to the control (Figure 5B). This confirmed the ability of IL-1β in promoting stemness/carcinogenesis in breast epithelial cells both normal and neoplastic.

## 4. Discussion

It has become clear that the mammary gland, an organ historically viewed as sterile, contains a specific microbiota, which may play important roles in the onset, progression, and dissemination of tumor cells [11,12,19,20]. This raises an important question on how these microorganisms can participate in breast carcinogenesis. Because *E. coli* was more prevalent in breast cancer tissues than in healthy controls [21], we decided to address here the pro-carcinogenic effects of breast microbiota through investigating the effects of *E. coli* on breast luminal and stromal fibroblast cells. We have shown that *E. coli* metabolites (E-CM) can activate breast stromal fibroblasts in a paracrine manner. Indeed, exposure of NBFs to E-CM increased the levels of several important markers of active fibroblasts namely, α-SMA, FAP-α, CD10, and GPR77. The two cell-surface molecules, CD10 and GPR77, were previously reported to define a CAF subset that is specifically correlated with chemoresistance and poor survival in patients with breast and lung cancer [16]. Furthermore, E-CM treatment enhanced the migratory/invasive and proliferative capacities of mammary fibroblasts, and at the molecular level upregulated the pro-migratory/invasive protein MMP-2. Matrix metalloproteinases (MMPs) are a group of endopeptidases essential for the breakdown of basement membrane (BM) and extracellular matrix (ECM) components. In breast cancer, the overproduction or enhanced activity of MMP-2 leads to the degradation of ECM and BM, enabling tumor cells to invade surrounding tissues and metastasize [17]. Importantly, we have found that *E. coli* metabolites activate the pro-inflammatory NF-kB signaling via p65 phosphorylation. This was confirmed by showing significant increases in the expression/secretion of three important pro-inflammatory/carcinogenic cytokines IL-6, IL-8, and IL-1β. In fact, the upregulation of IL-1b was tremendous (39- and 85-fold in NBF-6 and NBF-25, respectively) (Figure 1A).

NF-kB qRT-PCR arrays showed that E-CM modulates the expression of several other NF-kB target genes such as *CCL5*, *CXCL1*, *CXCL2*, *CFB*, *CSF3*, *VCAM1*, *STAT1*, and many others. This shows that E-CM promotes pro-inflammatory program in mammary stromal fibroblasts. Since active fibroblasts could be either myofibroblastic (high level of α-SMA) or inflammatory (high level of IL-6 and other inflammatory factors) [22], it seems that E-CM generates an inflammatory subset of active mammary fibroblasts. The other important cancer-promoting transcription factor STAT3 was upregulated at the mRNA level and activated at the protein level, confirming the induction of a strong pro-inflammatory response in primary mammary fibroblasts. Since active fibroblasts have paracrine pro-carcinogenic effects, we sought to test this on HMLE cells. Indeed, E-CM-treated fibroblasts promoted the proliferation, invasion, EMT, and stemness in HMLE cells. This further confirms E-CM-dependent activation of breast stromal fibroblasts, which could be mediated through the secretion of several pro-inflammatory/-carcinogenic factors. Similarly, *H. pylori* activated normal rat gastric fibroblasts, which promoted EMT in normal rat gastric epithelial cells [23]. It has been shown that *H. pylori* infection increased VCAM1 expression in gastric CAFs via activation of the STAT1 signaling pathway. VCAM1^+^ CAFs promoted gastric cancer progression and metastasis in vitro and in vivo via direct contact with gastric cancer cells [24]. In addition, VCAM1 secreted from CAFs enhanced the growth and invasion of lung cancer cells [25]. We have shown here strong upregulation of VCAM1 in NBF cells treated with *E. coli* secretome. Furthermore, it was reported that the *E. coli* cytotoxic necrotizing factor 1 (CNF1) toxin has the ability to induce EMT in human colon adenocarcinoma cells (HT-29) by upregulating the ZEB1, Snail1, and Vimentin, while showing de-localization of E-cadherin and β-catenin, activation of mTOR, enhanced rates of wound healing, and invasion [26].

In addition to the activation of mammary stromal fibroblasts, we have found that *E. coli* secretomes can also promote carcinogenesis in normal human luminal cells. Indeed, E-CM activated STAT3 and increased the proliferative and invasive capacities of HMLE cells, and promoted the EMT and stemness processes. This effect could be mediated through the secretion of several cytokines that were upregulated in response to *E. coli* metabolites. One of these mediators is IL-1β, which showed the strongest upregulation upon exposure of NBF cells to E-CM. This hypothesis was confirmed by showing that rhIL-1β can promote EMT and stemness features in normal breast epithelial cells (HMLEs) and ER-positive BC cells (MCF-7), through activation of the STAT3 pathway. This indicates that rhIL-1β can recapitulate the paracrine effect of E-CM-treated fibroblasts on breast luminal cells. In a previous study, it has been shown that rIL-1β promotes EMT and stemness through ZEB1 activation, and increases drug resistance in colon cancer cells [27]. This shows that IL-1β could promote both the onset and the progression of tumor cells.

## 5. Conclusions

Together, the present findings indicate that *E. coli* secretome can promote breast carcinogenesis through promoting EMT and stemness in mammary epithelial cells either directly, or indirectly through activation of stromal fibroblasts and the consequent paracrine pro-carcinogenic effects via secretion of several cytokines such as IL-1β.

## Figures and Tables

**Table 1:** The used primers.

Primers	Sequence	
IL-6	Forward	5′-AGACAG CCA CTC ACC TCT TCA G-3′
	Reverse	5′-TTC TGC CAG TGC CTC TTT GCT G-3′
IL-8	Forward	5′-GAT CCA CAA GTC CTT GTT CCA-3′
	Reverse	5′-GCT TCC ACA TGT CCT CAC AA-3′
GAPDH	Forward	5′-GAGTCCACTGGCGTCTTC-3′
	Reverse	5′-GGGGTGCTAAGCAGTTGGT-3′
IL-1β	Forward	5′-GCTGGAATTTGAGTCTGCCC-3′
	Reverse	5′-TCCACATTCAGCACAGGACT-3′

**Table 2:** Commonly upregulated NF-kB targeted genes.

Gene	NBF-6	NBF-25
*CFB*	24.8	33.4
*BIRC3*	3.3	15.8
*STAT1*	8.2	14.3
*IL1b*	6.5	9.8
*CCL11*	3.0	12.7
*CXCL1*	12.1	12.3
*CSF2*	11.8	12.2
*CXCL2*	9.6	10
*IL8*	4.7	9.8
*ICAM1*	10.9	7.9
*IL1A*	6.1	7.3
*CCL2*	4.1	7.2
*MYD88*	2.5	6.9
*NFKB2*	2.4	5.2
*IRF1*	2	4.9
*TNFSF1B*	2.2	4.9
*RELB*	2.8	4.9
*VCAM1*	9.6	4.2
*NFKB1*	2.1	3.6
*C3*	11.6	3.5
*CSF1*	2.6	3.4
*IL6*	6.1	3.3
*CSF3*	23.3	2.8
*NFKB1A*	3.1	2.7

## Data Availability

The datasets used and/or analyzed during the current study are available from the corresponding author on reasonable request.

## References

[1] Sung H., Ferlay J., Siegel R.L., Laversanne M., Soerjomataram I., Jemal A., Bray F. (2021). Global Cancer Statistics 2020: GLOBOCAN Estimates of Incidence and Mortality Worldwide for 36 Cancers in 185 Countries. CA Cancer J. Clin..

[2] Barbazan J., Matic Vignjevic D. (2019). Cancer associated fibroblasts: Is the force the path to the dark side?. Curr. Opin. Cell Biol..

[3] Tan Z., Kan C., Sun M., Yang F., Wong M., Wang S., Zheng H. (2022). Mapping Breast Cancer Microenvironment Through Single-Cell Omics. Front. Immunol..

[4] Hu D., Li Z., Zheng B., Lin X., Pan Y., Gong P., Zhuo W., Hu Y., Chen C., Chen L. (2022). Cancer-associated fibroblasts in breast cancer: Challenges and opportunities. Cancer Commun..

[5] Sahai E., Astsaturov I., Cukierman E., DeNardo D.G., Egeblad M., Evans R.M., Fearon D., Greten F.R., Hingorani S.R., Hunter T. (2020). A framework for advancing our understanding of cancer-associated fibroblasts. Nat. Rev. Cancer.

[6] Toumazi D., Daccache S.E., Constantinou C. (2021). An unexpected link: The role of mammary and gut microbiota on breast cancer development and management. Oncol. Rep..

[7] Urbaniak C., Gloor G.B., Brackstone M., Scott L., Tangney M., Reid G. (2016). The Microbiota of Breast Tissue and Its Association with Breast Cancer. Appl. Environ. Microbiol..

[8] Fu A., Yao B., Dong T., Chen Y., Yao J., Liu Y., Li H., Bai H., Liu X., Zhang Y. (2022). Tumor-resident intracellular microbiota promotes metastatic colonization in breast cancer. Cell.

[9] Nejman D., Livyatan I., Fuks G., Gavert N., Zwang Y., Geller L.T., Rotter-Maskowitz A., Weiser R., Mallel G., Gigi E. (2020). The human tumor microbiome is composed of tumor type-specific intracellular bacteria. Science.

[10] Cullin N., Azevedo Antunes C., Straussman R., Stein-Thoeringer C.K., Elinav E. (2021). Microbiome and cancer. Cancer Cell.

[11] Peters B.A., Kelly L., Wang T., Loudig O., Rohan T.E. (2024). The Breast Microbiome in Breast Cancer Risk and Progression: A Narrative Review. Cancer Epidemiol. Biomark. Prev..

[12] Hieken T.J., Chen J., Hoskin T.L., Walther-Antonio M., Johnson S., Ramaker S., Xiao J., Radisky D.C., Knutson K.L., Kalari K.R. (2016). The Microbiome of Aseptically Collected Human Breast Tissue in Benign and Malignant Disease. Sci. Rep..

[13] Banerjee S., Wei Z., Tan F., Peck K.N., Shih N., Feldman M., Rebbeck T.R., Alwine J.C., Robertson E.S. (2015). Distinct microbiological signatures associated with triple negative breast cancer. Sci. Rep..

[14] Hawsawi N.M., Ghebeh H., Hendrayani S.F., Tulbah A., Al-Eid M., Al-Tweigeri T., Ajarim D., Alaiya A., Dermime S., Aboussekhra A. (2008). Breast carcinoma-associated fibroblasts and their counterparts display neoplastic-specific changes. Cancer Res..

[15] Al-Mohanna M.A., Al-Khalaf H.H., Al-Yousef N., Aboussekhra A. (2007). The p16INK4a tumor suppressor controls p21WAF1 induction in response to ultraviolet light. Nucleic Acids Res..

[16] Su S., Chen J., Yao H., Liu J., Yu S., Lao L., Wang M., Luo M., Xing Y., Chen F. (2018). CD10(+)GPR77(+) Cancer-Associated Fibroblasts Promote Cancer Formation and Chemoresistance by Sustaining Cancer Stemness. Cell.

[17] Jiang H., Li H. (2021). Prognostic values of tumoral MMP2 and MMP9 overexpression in breast cancer: A systematic review and meta-analysis. BMC Cancer.

[18] Marotta L.L., Almendro V., Marusyk A., Shipitsin M., Schemme J., Walker S.R., Bloushtain-Qimron N., Kim J.J., Choudhury S.A., Maruyama R. (2011). The JAK2/STAT3 signaling pathway is required for growth of CD44(+)CD24(-) stem cell-like breast cancer cells in human tumors. J. Clin. Investig..

[19] Hieken T.J., Chen J., Chen B., Johnson S., Hoskin T.L., Degnim A.C., Walther-Antonio M.R., Chia N. (2022). The breast tissue microbiome, stroma, immune cells and breast cancer. Neoplasia.

[20] Chen J., Douglass J., Prasath V., Neace M., Atrchian S., Manjili M.H., Shokouhi S., Habibi M. (2019). The microbiome and breast cancer: A review. Breast Cancer Res. Treat..

[21] Urbaniak C., Cummins J., Brackstone M., Macklaim J.M., Gloor G.B., Baban C.K., Scott L., O’Hanlon D.M., Burton J.P., Francis K.P. (2014). Microbiota of human breast tissue. Appl. Environ. Microbiol..

[22] Li Y., Wang C., Huang T., Yu X., Tian B. (2023). The role of cancer-associated fibroblasts in breast cancer metastasis. Front. Oncol..

[23] Krzysiek-Maczka G., Targosz A., Szczyrk U., Strzalka M., Sliwowski Z., Brzozowski T., Czyz J., Ptak-Belowska A. (2018). Role of Helicobacter pylori infection in cancer-associated fibroblast-induced epithelial-mesenchymal transition in vitro. Helicobacter.

[24] Shen J., Zhai J., You Q., Zhang G., He M., Yao X., Shen L. (2020). Cancer-associated fibroblasts-derived VCAM1 induced by H. pylori infection facilitates tumor invasion in gastric cancer. Oncogene.

[25] Zhou Z., Zhou Q., Wu X., Xu S., Hu X., Tao X., Li B., Peng J., Li D., Shen L. (2020). VCAM-1 secreted from cancer-associated fibroblasts enhances the growth and invasion of lung cancer cells through AKT and MAPK signaling. Cancer Lett..

[26] Fabbri A., Travaglione S., Rosadi F., Ballan G., Maroccia Z., Giambenedetti M., Guidotti M., Odum N., Krejsgaard T., Fiorentini C. (2020). The Escherichia coli protein toxin cytotoxic necrotizing factor 1 induces epithelial mesenchymal transition. Cell. Microbiol..

[27] Li Y., Wang L., Pappan L., Galliher-Beckley A., Shi J. (2012). IL-1beta promotes stemness and invasiveness of colon cancer cells through Zeb1 activation. Mol. Cancer.

